# Cross-species virus transmission and its pandemic potential

**DOI:** 10.1186/s42269-022-00701-7

**Published:** 2022-01-25

**Authors:** Priyanka Ray Choudhury, Tapoja Saha, Sachin Goel, Janvi Manish Shah, Deepak Ganjewala

**Affiliations:** 1 Amity Institute of Biotechnology, Amity University Noida, Sector 125, Noida, 201303 India; 2 Department of Biotechnology, Noida Institute of Engineering and Technology, 19, Knowledge Park-II, Institutional Area, Greater Noida, 201306 India; 3 Department of Biotechnology, Thadomal Shahani Engineering College, Mumbai, 400050 India

**Keywords:** Pandemics, Zoonotic viruses, SARS-CoV-2, MERS, Epidemiology, Vaccine, ACE-2, COVID-19

## Abstract

**Background:**

The majority of pandemics are known to be a result of either bacteria or viruses out of which viruses seem to be an entity of growing concern due to the sheer number of yet unidentified and potentially threatening viruses, their ability to quickly evolve and transform, their ability to transfer and change from one host organism to another and the difficulty in creating safe vaccines on time.

**Main body:**

The present review attempts to bring forth the potential risks, prevention and its impact on the global society in terms of sociological and economic parameters. Taking hindsight from previously as well as ongoing current viral epidemics, this article aims to draw a concrete correlation between these viruses in terms of their origin, spread and attempts to compare how much they can affect the population. The study also assesses the worst-case scenarios and the amount of preparedness, required to fight against such pandemics and compares the required amount of preparedness to the current precautions and measures by different governments all across the world.

**Short conclusion:**

Learning from the current pandemic, we can implement certain measures to prevent the adverse effects of pandemics in the future and through severe preparedness can combat the challenges brought about by the pandemic.

## Background

Unlike epidemics whose scope of the outbreak is limited to only a certain limited area, a pandemic is a global disease outbreak. Throughout history, there have been many instances of such outbreaks which have resulted in the deaths of millions of people across the world in different periods. One of the most notable pandemics is known to be the Bubonic Plague (Black death) caused by the *Yersinia pestis* outbreak around 1346–1353. It wiped out as much as 1/3^rd^ of the European population (Duncan and Scott 2005). The plague continued to be both a public health threat and a biodefense concern. Recently, Rosario-Acevedo et al. (2021) have highlighted strategies important for plague mitigation and treatment.

**Fig. 1:**
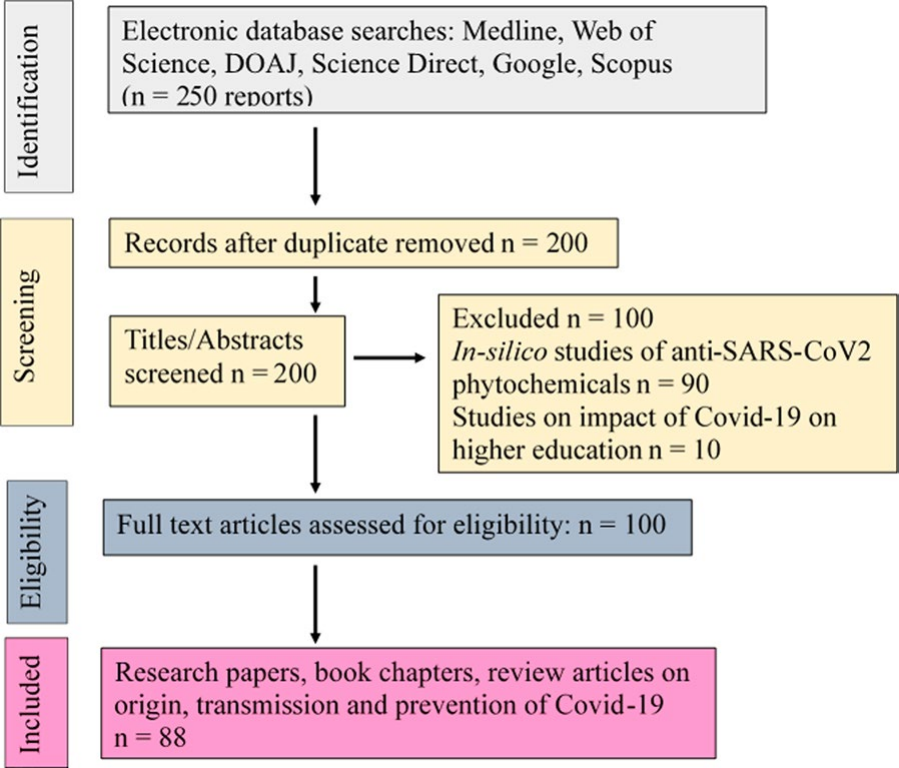
The PRISMA flow diagram of the literature review process

Any epidemic which spreads worldwide to become a pandemic poses an immense threat to the lives as well as the economy of the people. It may also lead to prejudice and amplify the undue preconceived notions toward certain communities as seen from the treatment of Jews (because compared to the rest of the population, the Jews weren’t affected as much) during the Black death and in recent times, the COVID-19 pandemic which has resulted in assaults of minority East Asian origin people residing in certain countries as their citizens (Gover et al. 2020). Recent reports have revealed that SARS-CoV-2 showed stability on glass, paper or wood, thus proper disinfection could be a crucial strategy to stop its spread. Various disinfectants, such as ethanol, isopropanol, sodium hypochlorite or hydrogen peroxide can effectively disrupt SARS-CoV-2. A comprehensive understanding of the SARS-CoV-2 infection, spread and multidirectional strategies can restrict the spread of the virus. Janik et al. (2021) have provided the most important information about SARS-CoV-2, such as its stability on different surfaces, protection strategies and decontamination options. The current COVID-19 pandemic is very similar to Spanish, Hong Kong, Asian and swine influenza pandemics as they all spread by the mobilization of people (Akin and Gozel 2020). Literature survey for the collection of relevant reports for this review is presented by Prisma flow chart in Fig. 1.

The causative agents of most pandemics (as well as potential pandemics) are often bacteria or viruses. The virus pandemics are generally caused by influenza viruses; the problem with such viruses is that they have the capability of changing with the change in season. The rapid change in their structure means that the general population doesn’t get to develop immunity against them naturally (Pike et al. 2010). Despite all predictive techniques, the virus tends to change its behavior unpredictably, and thus, the ever-changing and rapidly evolving viruses, coupled with the issue that creating antiviral drugs and vaccines is difficult (due to the ever-changing viral structure itself and many other challenges) pose a great threat as potential pandemics (Santos and Monteiro 2013). Bacteria, on the other hand, pose a different kind of threat due to the increasing resistance to antibiotics. Epidemiological studies have shown a close link between antibiotic consumption and the emergence and dissemination of resistant bacteria strains (Anonymous). In the food industry, bacterial resistance emerged because the overuse of antibiotics has led to many different strains, which need even stronger antibiotic drugs to combat the disease (Ventola 2015). With the advancements in technologies, it has become very easy for individuals to transmit a disease from one part of the globe to another whether knowingly or unknowingly. Thus, it has become relatively easier for any infectious and deadly disease/epidemic to potentially become a pandemic.

## Main text

### Discovery of new viruses and modes of transmission

There is a vast multitude of viruses already in different species of animals that may or may not be identified as of yet; out of these viruses, there may be viruses that may be capable of getting transmitted into human beings and causing ill effects. Viruses that originate and transmit through such a mode are known to be zoonotic viruses, and the mode of transmission is known as cross-species transmission (Jonas and Seifman 2019). Generally, viruses do not possess the capability of being able to infect the newly acquired human host (in this case since humans are the species of main concern) that wasn’t previously susceptible or had been previously exposed therefore, such transfers need to be coupled with increased exposure and attainment of sufficient variations within their structure which would make it possible for them to overcome the cellular and immunity barriers and allow infection of the new host (in this case, human beings) (Liu and Saif 2020).

**Fig. 2:**
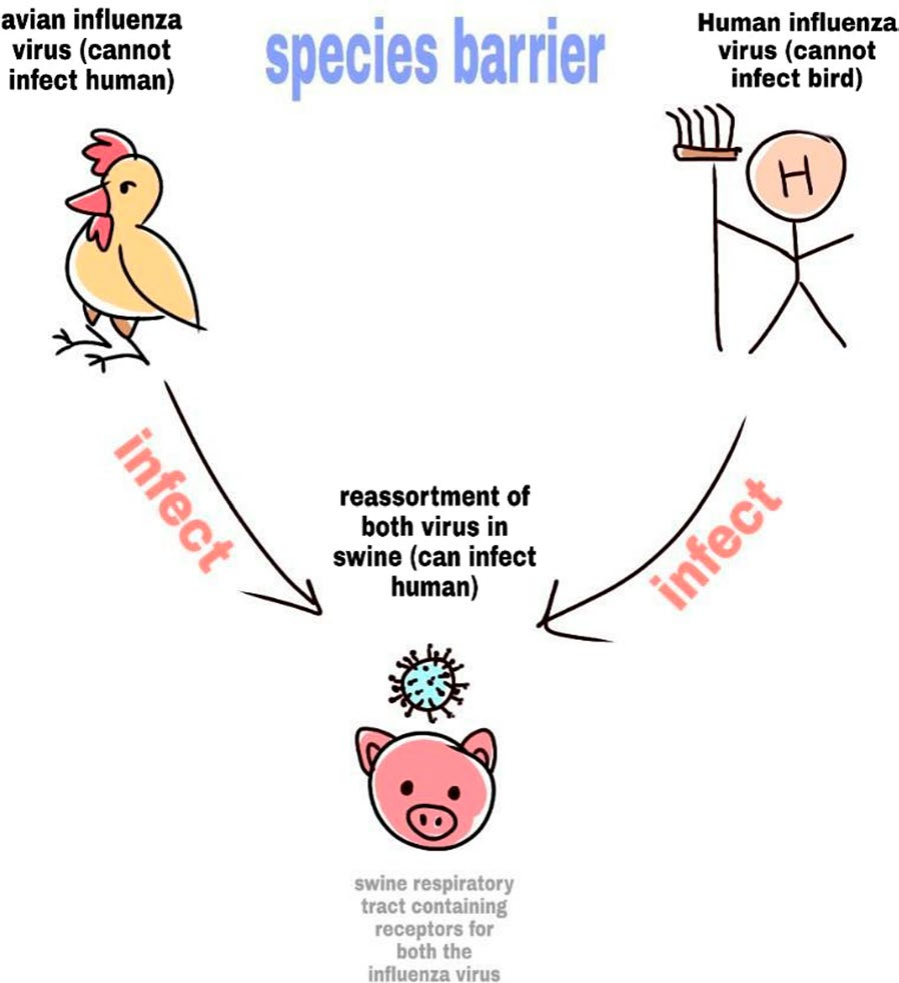
Re-assortment of different influenza viral strains in intermediate organisms

The scenario of cross-species transmission may be explained simply by using the example of the 1918 flu pandemic. It is said to have originated in a farm where a bird with the flu virus and a human with another flu virus came in contact with a pig. The virus in the bird couldn’t affect humans and vice versa however, they both could infect the pig. Inside the cells of the pig host, the two flu viruses through re-assortment, form a new strain of H1N1 (avian origin) which was able to infect humans; thus it is impossible to predict all the ways a virus may evolve. The following example demonstrates the “mixing vessel” hypothesis. Since pigs are susceptible to influenza infection from the bird (avian) as well as a human reservoir, it is hypothesized that both these viruses are capable of co-infecting the pig and within which, re-assortment can take place as shown in Fig. 2 (Ma et al. 2010). This hypothesis was first brought forward by Scholtissek and his colleagues in 1985. It was thought that Influenza A viruses do not usually spread from avian sources to humans and vice versa easily; Due to the presence of a low species barrier in pigs, it is hypothesized and tested to be true that both the avian and human viruses are successful at infecting swine (Scholtissek 1990). The swine respiratory tract possesses both specific receptor types which have SAα2,3Gal (avian) receptor and Saα2, Gal (mammal) receptor. These receptors are the preferential binding sites for avian and mammalian influenza viruses, respectively; since pigs contain both these receptors, it is said to be an ideal “mixing vessel” for such viruses (Ma et al. 2009). Other studies do not confirm the re-assortment vessel of the Spanish flu to be swine but suggest there may be some other intermediate involved since there is no immediate proof for the intermediate host to be pig as suggested by Antonovics et al. (2006) and Gibbs and Gibbs (2006) (Gibbs 2005). Since the zoonotic transmission is rarely a one-way transmission, cross-transmission from either species to one another can thus take place effectively (Mole 2013).

There has been a lot of contradictory studies challenging the above notion. In the review by Greger (2006), it wasn’t possible to clarify whether the pigs infected the re-assorted virus to humans or humans infected the pigs with the already effective virus (avian source). After genomic analysis of the 1918 flu virus, Taubenberger et al. (2005) made a proposition that instead of being a reassortment virus, it was more likely a virus of avian origin that directly crossed the species barrier to humans without any external aid of an intermediate organism (Taubenberger 2006). However, it is commonly believed that the 1957 (Asian flu) and 1968 (Hong Kong flu) viruses were re-assortment viruses meaning, there might be an intermediate involved in the change and transfer of these viruses into the human population (Kilbourne 2006).

From the above scenario, it is clear that animal farms, especially with multiple types of animals are an excellent place for new virus strains to brew especially due to the abundance of different species of animals in close contact. In the instance, wild animals are surrounding the farm, it only increases the chances of more exposure to new viruses. Modern farming has also brought each domesticated animal close to each other, pumped with antibiotics (to simply keep them alive till they’re ready for consumption) which may not only lead to newer viruses emerging but also lead to antibiotic resistance to the excess antibiotics being stored in the fatty tissues of these animals (Delabouglise et al. 2020). Other sources may include newly inhabited areas where the newly pioneered people and their domesticated animals come in contact with the other wild fauna of the area (Stevenson et al. 2013).

Wet meat markets are yet another source from where new novel virus strains may develop and spread. It is imperative to understand that these viruses already exist in nature but are usually unable to transmit to humans due to species barriers. However, places such as wet meat shops contain large amounts of animals of different species which are killed freshly. The different species of animals for consumption are kept in close contact to save space thus making it a great hotspot for “mixing” and re-assortment of various viruses which otherwise couldn’t infect human beings (Woo et al. 2006). The current pandemic of COVID-19 is said to have originated on such markets in Wuhan province, China which contains a wild animal market along with the wet meat markets thus making matters worse (Li et al. 2006a, b). It is also proposed that climate change and melting of the polar ice caps could potentially lead to exposure of many ancient viruses that were previously buried under the snow and pose a future threat (Houwenhuyse et al. 2018).

## Coronaviruses

**Fig. 3:**
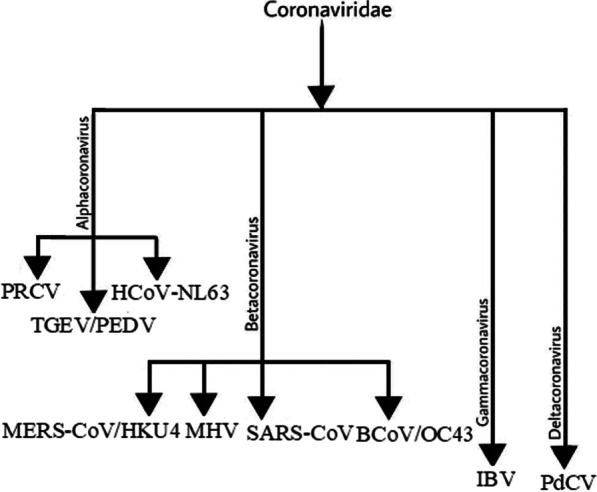
Genera Alphacoronavirus including viruses that can infect human population example Including human coronavirus (HCov-NL63), porcine respiratory coronavirus (PRCV) and porcine transmissible gastroenteritis coronavirus (TGEV) and Betacoronaviruses which includes viruses that can infect human beings e.g., Bat coronavirus HKU4, SARS-CoV, mouse hepatitis virus (MHV), human coronavirus (OC43), bovine coronavirus(BCoV), and MERS-CoV. Gammacoronavirus includes viruses that are capable of infecting the avian population whereas Deltacoronavirus comprises viruses that can effectively infect mammalian as well as avian species e.g., Including infectious bronchitis coronavirus (IBV) and procrinedeltacoronavirus (PdCV), respectively

First identified in Guangdong province in Southern China (2002 November), SARS-CoV is said to be a virus of unidentified animal origin (suggested to be of civet or bat origin). Its outbreak occurred in 26 countries worldwide. The aforementioned area is now termed as one of the areas of re-emergence of SARS (Xu et al. 2004). Commonly known as a severe acute respiratory syndrome-related coronavirus (SARS) is a mammalian virus that can successfully infect certain mammals notably humans and bats. Coronaviruses belong to the family Coronaviridae; Order Nidovirales (Fehr and Perlman 2015; Satyam et al. 2021). Coronaviridae is further subdivided into four genera and has seven types of viruses namely, HCoV-SARS, HCoV-NL63, HCoV-OC43, HCoV-HKU, HCov-299E, MERS-CoV, SARS-CoV, and SARS-CoV-2 (Fig. 3).

The SARS coronavirus is a positive-stranded RNA virus (Satyam et al. 2021). Among all other RNA viruses, coronaviruses are known to possess the largest genome having a capacity ranging from 27 to 32 kb usually. The packaging of the genome is within a nucleoplasmid (N) protein containing a helical capsid. It is further surrounded by an envelope (Hui et al. 2004). A typical coronavirus has three crucial structural proteins associated with its viral envelope, envelope protein (E), spike protein (S), and membrane protein (M). Membrane and envelop proteins are mainly involved with viral assembly whereas S is involved in viral entry into the host organism’s cells. The spike is considered to be a member of the class I viral member fusion proteins. These spikes, similar to other coronaviruses undergo proteolysis during the later stages of cell entry/invasion processes (sometimes after receptor binding); the conformational changes in the spikes are notably different from other class I membrane fusion proteins (as noted in many coronaviruses). The proteolysis during the later stages results in a direct membrane fusion (low pH and receptor binding also playing a crucial role in the fusion) (Ksiazek et al. 2003).

The SARS coronavirus contains two main subdomains, which are receptor binding motif (RBM; two-stranded antiparallel β sheets) and core structure (which comprises five stranded antiparallel β sheets). At the time of the SARS outbreak, SARS-CoV strains of a high degree of similarity were identified and isolated from patients and animals from markets; these strains’ S1-CTDs differed in receptor-binding motif (RBM) region by two residues only-Thr487 and Asn479 (humans) and Ser487 and Lys479 (civet viral strain) both of which recognize zinc peptide angiotensin-converting enzyme 2 (ACE2) however, the binding affinity of the human SARS-CoV S1-CTD for ACE2 is higher. It is to be noted that neither the ACE2 mediate any role in the viral entry, nor does the binding of SARS-CoV interfere with ACE2’s enzymatic activity. ACE2 protein consists of ~ 805 amino acids, of which 723aa encodes a large extracellular domain also called ectodomain, 21aa a transmembrane domain and 44 aa a short cytoplasmic domain also called endodomain (Tipnis et al. 2000). The ectodomain of ACE2 protein encompasses a membrane-proximal collectrin and membrane-distal peptidase domains (Li 2015).

**Fig. 4:**
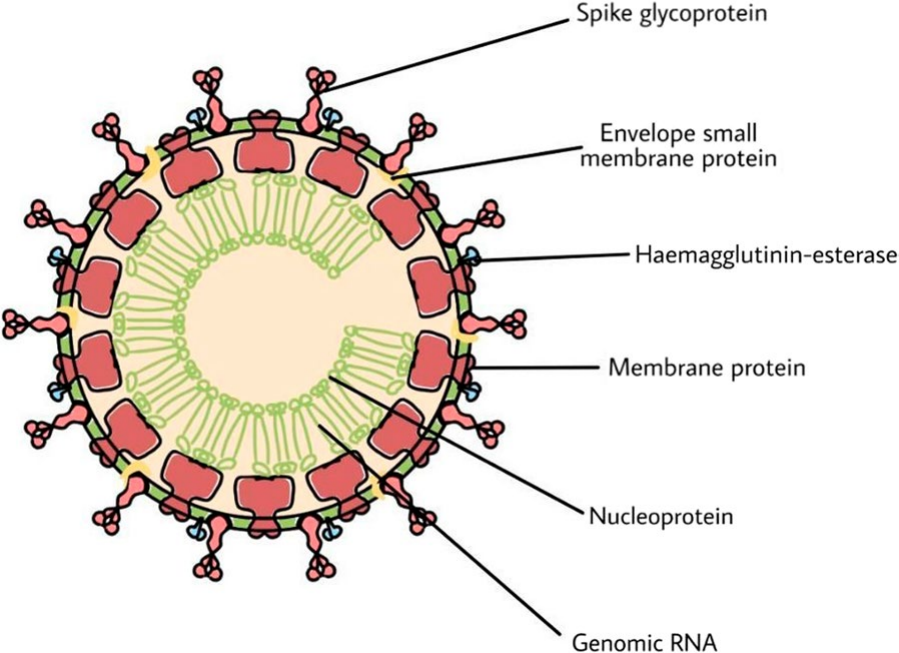
Structure of novel SARS-CoV-2 (COVID-19 coronavirus)

SARS-CoV-2 (earlier called 2019-nCoV) is the causative agent of the coronavirus disease 2019, also known as COVID-19. It is a zoonotic virus having a genome size of 29,891 nucleotides, which is structurally similar to Betacoronaviruses like SARS-CoV (2003) with a high mutation rate and recombination capacity (Mousavizadeh and Ghasemi 2020). It has a crown-like appearance with pleomorphic or spherical form and diameters may range from 60 to 140 nm (Fig. 4). It is sensitive to heat and radiation and can be inactivated by lipid solvents such as ethanol (Chen et al. 2020). It is a single-stranded RNA virus containing a right-hand RNA dependent RNA polymerase (RdRp; also called nsp12) domain along with an N terminal extension domain that is unique to nidoviruses. The RdRp works in conjunction with nsp7 and nsp8 (co-factors) and plays a key role in the transcription and replication processes of the virus) (Marra et al. 2003; Peiris et al. 2003). The N terminal domain can adopt a nidovirus RdRp-associated architecture of nucleotidyltransferase (NiRAN); an interface domain connects the aforementioned domain with NiRAN. Having a multi-subunit replication mechanism, it contains a set of nsps i.e., non-structural proteins (resultant of a cleavage product of ORF1ab and ORF1a viral polyproteins which facilitate the replication and transcription process) (Li et al. 2003 2006a, b).

There are various structural similarities between SARS-CoV-2 and SARS-CoV viruses having a similar nsp12-nsp7-nsp8 complex. Their dissimilarity may be due to differences in a few residues in the nsp12 and NiRAN domains. These domains also contain some additional residues constituting a structural block consisting of two helices and five antiparallel β strands. In the RdRp domain, an additional N-terminal β hairpin gets into the groove clamped by the palm subdomain and NiRAN domain (Bafna et al. 2020).

The spike structure is similar to SARS-CoV; these spikes lock onto the proteins on the cell surface and also has a binding affinity to ACE2 (zinc metalloprotease); infecting the target cells by binding to ACE2 through surface spike protein and modulating the expression of ACE2 (Beniac et al. 2006; Kirchdoerfer et al. 2016; Walls et al. 2016).

## Origin of SARS-CoV-2

Eco health alliance is an NGO that has been trying to identify various unknown viruses in Southern China. They have been examining bats in caves, scanning and testing them for unknown viruses and flagging the ones that could potentially infect humans. The identified viruses are then labelled as high risk or low risk depending on their similarity to already known infectious viruses. It is proposed that bat coronavirus RaTG13 which was previously classified into the “low risk” category might have evolved into the now known virus due to its staggering 96% identity; it is believed that the initial virus may have mutated in another bat or perhaps jumped into another species before jumping to human beings (Sahin 2020). This animal to human jumping of the virus is known to have occurred in Wuhan (Hubei province of China) in December 2019.

## Epidemiology

For SARS viruses, based on the analysis of 1425 cases on 28th of April in Hong Kong SAR by Donnelly and his colleagues, the maximum estimate of the likelihood of mean came out to be 6.37 days, and the variance of time taken from infection to the onset of disease was around 16.69 days (Table 1). Thus, the predictive time for the onset of disease after infection was calculated at 14.22 days, meaning that it is expected for the symptoms of the disease to be visible within the stipulated amount of time (Lee 2003). The estimation is presented in Table 2.

**Table 1:** Estimated time of incubation as per WHO consensus

	Time of incubation
Mean	4–6 days
Median	4–5 days
Minimum	1
Maximum	14

**Table 2:** Estimated incubation period (days) by geographical region (WHO 2003)

The incubation period (Days)				
Area	Minimum	Maximum	Mean	Median
China	1	12	4	4
Vietnam	5	10	6–7	–
Singapore	1	10	5.3	5
Europe	5	10	7.2	7
Canada	2	10	4.8	4.2

The efficacy of transmission of the SARS viruses is at its peak during the first two weeks of the infection i.e., the period of severe illness after the onset of the period of continual medical deterioration. The transmission efficiency gradually decreases with the passage of time and recovery (Meltzer 2004). Route of transmission is mostly through direct contact of viral droplets and entry into the body via eyes, nose and mouth by a person to person transmission.

The incubation time of the SARS-CoV-2 between exposures to onset ranged 1–12.5 days with the median estimates of 5–6 days. The maximum period of incubation is said to be around 14–15 days. The transmission can occur at any time from even an asymptomatic person who may be tested positive for the virus. The most affected individuals and individuals at high risk are healthcare workers (due to prolonged exposure) and people of age/people with underlying disease conditions (Byrne et al. 2020). Since it can transmit from person to person more effectively and quickly than SARS, it is estimated being at a close distance of 1 m with an infected individual is enough for the droplets to enter the body of the new host. The pathway of entry is similar to SARS i.e., via eyes, nose and mouth. The virus is also capable of transmission through inanimate objects as the droplets containing viruses remain viable for varying periods depending on the surrounding environment.

A recent review has summarized different types of acute respiratory diseases and briefly discussed earlier outbreaks of coronaviruses and compared their occurrence and pathogenicity with the current COVID-19 pandemic (Khan et al. 2020). Also, the review shed light on various epidemiological aspects, different technical issues, patient management strategies related to COVID-19 and different strategies for the development of effective vaccines and therapeutic combinations to deal with this viral outbreak (Khan et al. 2020).

## Case fatality ratio

**Fig. 5:**
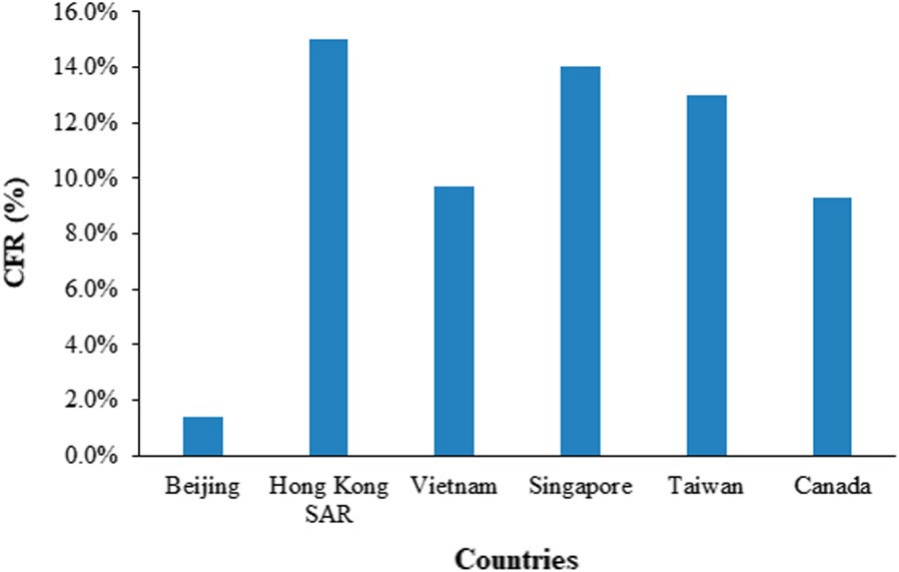
Crude case fatality ratios area-wise (According to WHO consensus)

**Fig. 6:**
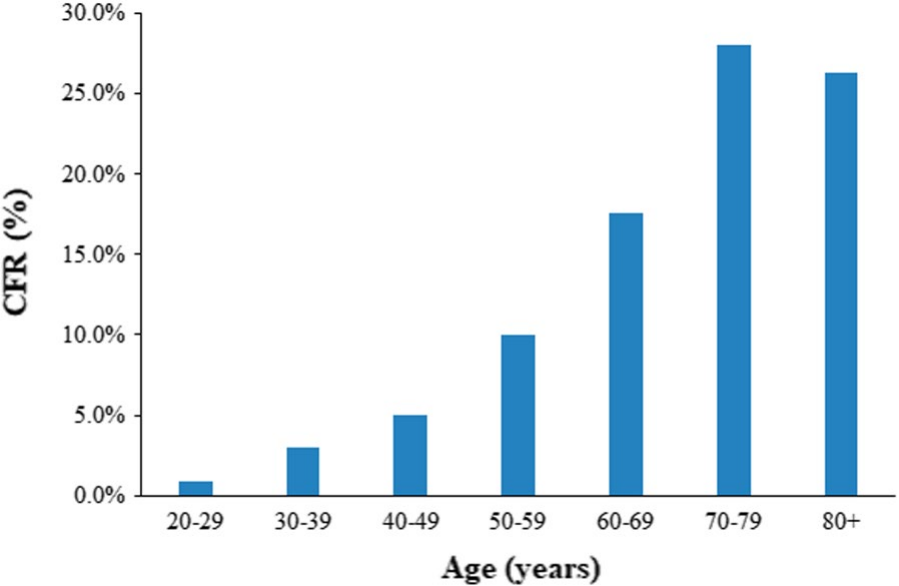
Age-specific CFR (As per WHO consensus)

Depending on the age of the patient, the case fatality rate (CFR) for SARS may range from 0 to 50%; with the help of statistical methods, the CFR was measured to be around 15% in Hong Kong SAR followed by 14% in Singapore (Fig. 5). It was also noted to be more lethal in the aged and elderly, people with underlying disease conditions and male gender (Fig. 6). The overall death rate was calculated at 4% globally initially, however after the outbreak ended it has been observed that the death was around 9.55% (Jia et al. 2009).

**Fig. 7:**
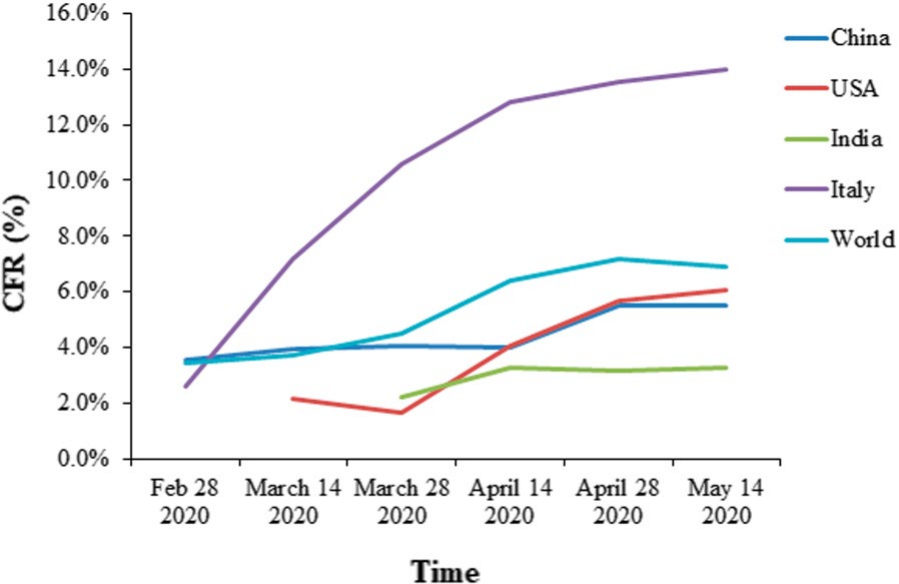
The case fatality rate (CFR) from Feb-March 2020 (as per ourworldindata.org). The CFR rates may vary according to the methodology used for calculation as well as the source of data taken

**Fig. 8:**
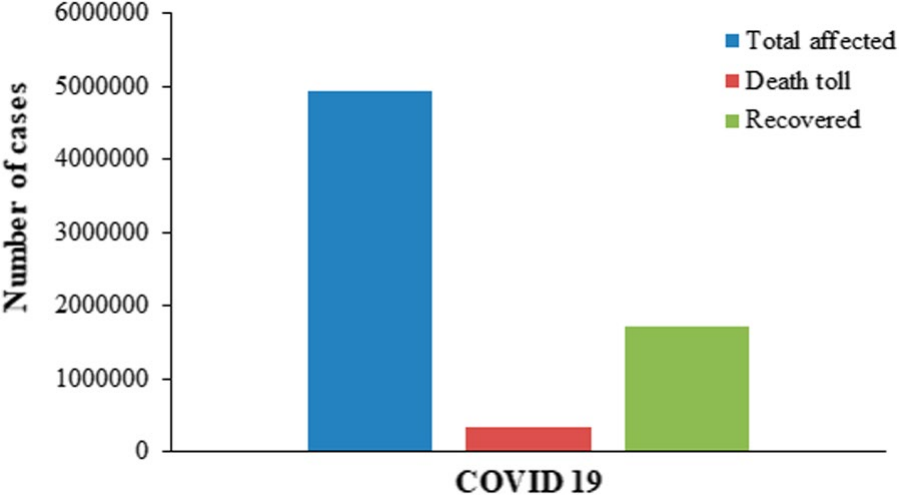
The total affected versus dead versus recovered patients of COVID-19 (as of 20th May 2020)

As observed in the case of SARS, COVID-19 is known to be more lethal in the aged population, people with underlying health issues and men (due to higher concentration of ACE2 in men’s bloodstream (confirmed) and lower immune system as compared to women (unconfirmed)) (Chen et al. 2014; Costa et al. 2003; Cui et al. 2020). The CFRs currently vary from country to country with Italy/USA having the highest during the first wave as shown in (Figs. 7, 8).

**Fig. 9:**
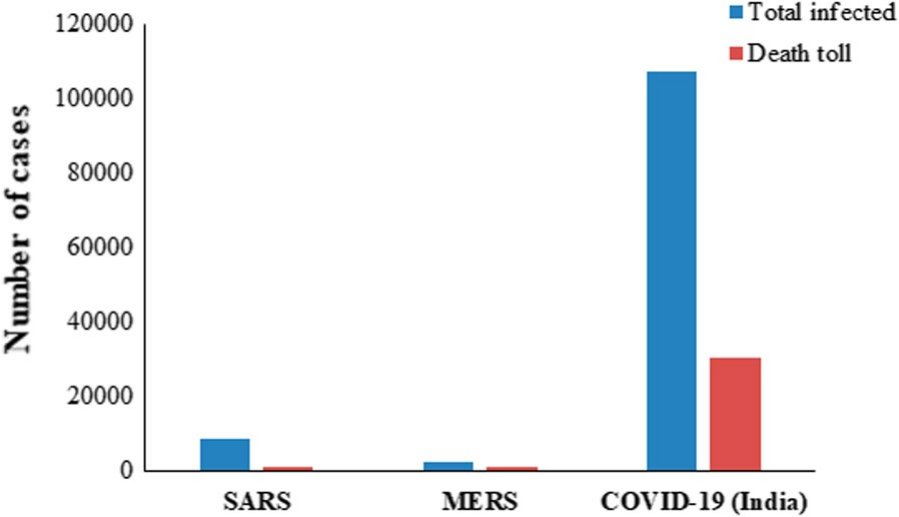
Comparison between SARS, MERS and COVID-19

**Fig. 10:**
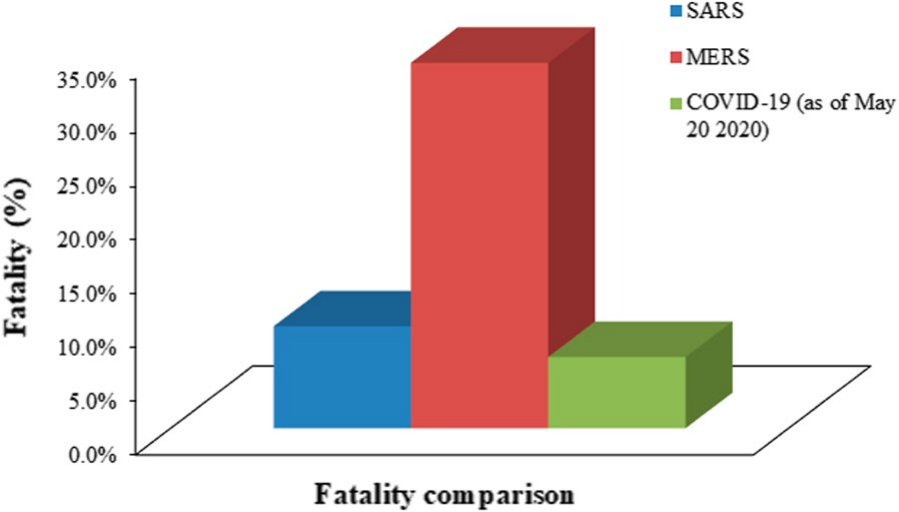
Comparison of fatality

To become a pandemic, the causative agent (usually virus or bacteria) must possess a good balance of contagiousness and deadliness. The total estimated death toll is presented in Table 3 and comparative statistics in Figs. 9 and 10.

**Table 3:** Comparison of estimated death tolls of pandemics (the numbers may vary from site to site). *Sources*

Pandemic	Year	Death count	Notes
Antonine Plague	165 AD	5 million	The cause is yet unknown although it is thought to have been measles or smallpox
The Black death	1346–1353	75–200 million	Lead to mass deaths and devastation across three continents. Causative agent: *Yersinapestis*
Third Cholera pandemic	1852–1860	1 million	Considered to be the most deadliest of all the cholera pandemics; Originated in India
“Russian flu”	1889–1890	1 million	Causative agent: Influenza A virus subtype H3N8
Sixth Cholera pandemic	1910–1911	800,000 +	–
“Spanish flu”	1918	20–50 million	Affected young and healthy individuals; infected over a third of the world’s population
“Asian flu”	1956–1958	2 million	–
SARS	2002–2003	774	–
MERS	2012	912	–
COVID-19	2019–2020	324,000 +	As of 20th May 2020

## Impact of pandemics

### Social level

Quarantine methods are applied to contain the disease as it is known to have a certain degree of effectiveness in preventing the spread of the disease, however it also means that a lot of people might get stuck in various places where they might not belong, for instance, people on trips, travels, ships, etc., might get stuck and would need to get back to their respective homes. Since their returns would require a lot of travel, it would be difficult and risky (Lemon et al. 2005).

In many poorer sections of society, it is often observed that people move from their villages to the cities in search of jobs and occasionally go back to their village to send the money. As observed in many parts of India during the current pandemic, these laborers aren’t able to return to their homes and are stuck. Such problems require a swift and efficient evacuation process by the respective governments. Also, it is advisable to avoid any non-essential long-distance travel as soon as a pandemic is declared even if it hasn’t reached one’s country yet (Alam and Zaini 2021).

A lot of individuals who live alone may start suffering from loneliness and may or may not observe a decline in their mental health. People with depression and/or other mental illnesses may have a harder time irrespective of whether they’re staying in the quarantine alone or not (Torales et al. 2020).

In abusive households, since the abuser would now have to spend an extensive amount of time indoors, the instances of domestic violence and child abuse may increase. Depending on the area of origin of the disease, there may be a rise in racism and violence against the people belonging to the area (or having originated from that area). There have been cases of violence against Chinese and other Asian people all across the globe after the COVID-19 outbreak.

The second wave of COVID-19 is now a past phenomenon in most of the countries, the curve of infection rises yet again and if not contained within a stipulated amount of time, might lead to effects more adverse than that of the first outbreak. These effects are more pronounced in some cases due to an already weak economy due to the first lockdown and already exhausted resources due to previous infections. If not planned well, reopening everything after the first lockdown can prove to be more dangerous especially for nations with less power (Ranjan et al. 2021).

### Economic impact

If the disease affects a sufficient number of young adults, it could lead to a substantial loss in not only the youth years of the individuals but also the amount of workforce. The sharp decline in economic exchange might occur which is often a result of protective measures such as quarantine.

Due to the shutdown of various small businesses, a lot of people lose jobs and livelihoods. Local producers are not able to sell their goods. While small businesses are affected tremendously, high ranking individuals of bigger companies remain more or less stable during such times (Bartik et al. 2020). Certain industries might cut-off payments and fire people to avoid any harm to their paycheck, others might fire their employees as they genuinely can’t afford to pay, some might not fire their employees and put them on unpaid leave while some businesses might simply collapse (Pak et al. 2020). Due to the lack of circulation of money, there might occur a significant gap between the rich and poor or might expand the already existing gap between them (Blustein et al. 2020).

People filing for unemployment might get paid higher than healthcare workers with millions of people filing for unemployment benefits during the first wave in America. Major suffering to small owned by women of color and minorities and 6.2% unemployment as of February 2021. In India, a 27.11% unemployment upsurge in the first wave was seen, and a 6.98% upsurge in the second wave occurred. A lot of resources and money which was allocated to the marginal communities of the society might get unfairly transferred to other sectors of society as governments might display a bias. The stock market may get adversely affected as people frantically sell out their stocks in an attempt to not incur losses with the progression of the disease.

The negative impact on the economy may get aggravated by risky behavior of individuals by mass gathering due to personal interests, religious matters or protests which serve as super spreader events, if not controlled in time, it might lead to a high degree of spread across the society. It may also occur from misinformation about the fatality and contagiousness on account of which individuals may ignore the government warnings and stop taking adequate measures. Misinformation and selective bias might prove to be fatal for all individuals (Kumar et al. 2020a, b).

Although the overall economic damage might not be avoidable completely, the adverse effects to the marginal communities can be counteracted by microfinance institutions for low-income families (Malik et al. 2020) and social welfare help in the form of food and necessities donations. Another adversity faced could be an unequal distribution of resources which is highly likely to occur in poorer economic strata, this could lead to a higher spread in poorer countries if they have a higher population of individuals who cannot afford proper treatment or isolation (Jensen et al. 2021).

Bilateral deals made by richer countries for the development of vaccines at a faster rate might lead to the formulation of a vaccine faster, but it also means that the supply will be shipped to those nations first, often putting the poorer and more affected nations at the back-burner (Callaway 2020). These richer nations might over-order the dosages to ensure that they can cover their populations twice as a safety measure but this only delays the supply to poorer nations as some poorer nations are estimated to reach vaccination status by the year 2023 which is far behind the richer nations already receiving their second dosages (Wouters et al. 2021).

### Based on age

The COVID-19 disease is known to be highly fatal in the aged population leading to widespread ageism where elderly patients are denied equal treatment-medically as well as socially. This is not just in the case of diseases such as COVID-19 where the aged population make up the high-risk fraction, even in other outbreaks, there have been instances of preferential treatment of younger individuals as compared to aged individuals (Fraser et al. 2020).

This is mainly due to the common misconception that if the life expectancy is around 81–82 years, an individual who is 80-years-old has approximately 1–2 years to live. The above notion is false because during the calculation of life expectancy, what lowers the age of expectancy is people who die young. An individual who has successfully reached 80 years of age has about 8–10 more years to live on average.

Due to excess patients and lack of ventilators, the equipment was being moved from the older patients to the younger ones. Even if it is due to the need of the hour, it is not a good practice, and such situations can be avoided by preventive measures and by having adequate medical preparedness for such situations. If the pandemic is well controlled, each individual shall be able to have equal access to health irrespective of income or age (Iyengar et al. 2020).

## Preventive measures and challenges

### Preparedness of medical sector

**Fig. 11:**
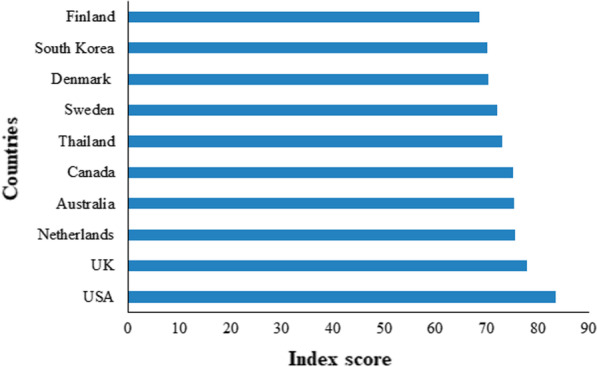
The top 10 countries are best prepared for a pandemic

It is imperative to note that simply having a good medical facility does not suffice in controlling a pandemic/epidemic. It is important to have adequate caution, effective social measures as well a strong conscience to avoid the spread of the disease. The statement above can be truly validated by the fact that despite having the best preparedness among all other countries, Despite the chart some of the richer countries somehow managed to perform poorly by having some of the highest rates of COVID-19 patients and transmission (Kumar et al. 2020a, b). The list of countries that are known to be well prepared for a pandemic/epidemic is depicted in Fig. 11.

During times of pandemics, it is very easy for the hospitals to get over-flooded with more than a usual number of patients and the medical staff getting overworked and overburdened. In countries with an already shortage of medical staff and admission beds, this can be a big issue (Bhuyan 2021). Also, since the medical workers, doctors, etc., are more likely to be in continuous direct contact with the patients, they can get infected as well. This may lead to a further shortage of medical workers and aggravate the deterioration of the situation (Bajpai 2014). There need to be adequate testing kits for the early detection of the disease so that its further spread can be avoided.

### Availability of PPEs

As multiple people get infected across the world, it is necessary to have an adequate number of personal protective equipment for the infected individuals and healthcare workers; this is necessary to prevent the spread of disease to healthy individuals and protect the medical/healthcare workers who are treating the patients. As observed from the current pandemic scenario of COVID-19, there seems to be a shortage of supply of necessary PPEs to the required sectors with the shipment being diverted to various other sectors (Ranney et al. 2020).

### Safety of healthcare workers

The working conditions must be safe, and mass testing needs to make sure that the safety of healthcare workers should not be in jeopardy. Through quarantining and minimizing contact, human to human transmissible diseases can be prevented (Lopes-Júnior et al. 2020). Certain other diseases that spread via zoonotic sources or contaminated food, water, etc., need special care in the sanitation and cleanliness department. Incentives could be awarded to the healthcare workers for the extra work done during the outbreak. It is important to cater to the medical needs of the infected while at the same time ensuring that the safety of healthcare workers is maintained. However, in cases of poor management, the medical workers may catch the illness and if adequate testing is not done, they may accidentally spread it further. Special care needs to be taken for the disposal of medical equipment that may be contaminated and infectious, the hygiene and sanitation within the clinics, and hospitals need to be optimum (Portoghese et al. 2014).

### Herd immunity

The phenomenon of herd immunity is achieved in a population when the maximum number of people in that population develops resistance to a certain disease through exposure or more commonly, vaccination. There are two possible ways to achieve herd immunity. Vaccination is the easiest and fastest way to achieve herd immunity and leads to fewer death counts. However, if the disease does not have any known vaccine (i.e., if it is a disease caused by a new viral strain) then the process of vaccine development may end up taking a lot of time meanwhile, the disease might continue to infect people. Another way to achieve herd immunity is to allow people to get exposed to the disease. The surviving people may develop immunity against the disease. However, a lot of people would die in the process (John and Samuel 2000).

The second method is a highly risky one because if the fatality of the new disease isn’t well known and the disease turns out to be highly contagious as well as highly fatal, then the maximum population would get wiped out before anyone could develop any immunity. Another reason why the second method might not work too well is that often, the viral strains mutate too quickly. Any resistance or immunity that may result from the infection may last for a limited amount of time (Fine et al. 2011).

HCoV-OC43, HCoV-229E, HCoV-HKU1 and HCoV-NL63 are some successful coronaviruses that cause flu and common cold from time to time. Although it is possible to fight off the infection caused by them, the acquired immunity lasts for only around 1–2 years. These viruses may also transform into a new virus once they get into the human population (Randolph and Barreiro 2020).

### Vaccines

As mentioned earlier, they are the simpler solution to achieve herd immunity however, the only issue is that it's not simple to develop a new vaccine from scratch. A typical vaccine production goes through several stages followed by multiple testing, clinical testing for safety, etc., before it is finally deemed as fit for public use. Often it contains inactivated or weakened microorganisms of the causative disease or sometimes its proteins and surface antigens to trigger the production of antibodies against the disease in the body. It takes a minimum of 18 months or sometimes even years to come up with a safe and effective vaccine.

Even if the developed vaccine is safe, sometimes the individual’s body does not react effectively to it (meaning: doesn’t produce the sufficient amount of antibodies to prevent the disease). Although there may be various reasons for that (such as age, initial immunity, low count of β cells to name a few), and it can be overcome through various measures (such as booster doses, adjuvants, etc.), it may still pose a challenge if the inefficacy goes unnoticed in time which generally means that the individual would still develop the disease despite having had the vaccine for it.

## Strategies for the effective management of the pandemic

### Roles of current antiviral drugs and vaccines

At the present, massive number of compounds are being tested and reported in the literature for the treatment of COVID-19. The history and current knowledge on drugs and therapeutic agents including antivirals like chloroquine, ivermectin, nitazoxanide, hydroxychloroquine, lopinavir, remdesivir, tocilizumab, supporting agent vitamin C, vitamin D, azithromycin, corticosteroids and promising investigational vaccines have been discussed elaborately (Dos Santos 2020). Based on certain inconclusive studies and research, desperate measures were incorporated in the form of resilient drugs like Hydroxychloroquine and Remdesivir, which however were not of much significance in combating the pandemic (Khuroo 2020). A recent report showed that hydroxychloroquine treatment significantly lessens viral load/disappearance in COVID-19 patients if given with azithromycin (Gautret et al. 2020; Aouissi et al. 2021). A study by Grein et al. (2020) supported the compassionate use of remdesivir for clinical improvement in COVID-19 patients. Remdesivir in a combination of baricitinib is more effective than remdesivir alone in speedy recovery of patients with Covid-19, receiving high-flow oxygen or noninvasive ventilation (Kalil et al. 2021). Several vaccines have been produced all across the world at record-breaking times to combat the disease before it gets out of hand (Lurie et al. 2020; Moore and Klasse 2020). They may be of several types as: (1) attenuated vaccines containing a weakened form of viruses; this virus is recognized by the body and triggers an immune response without infecting the body (Minor 2015). This contained a weakened form of viruses, which is made to mutate its genome to a point when it is unable to cause diseases. This is achieved by making them pass through animals or humans. The attenuated virus works by mediating strong T-cell and B-cell responses by replicating a natural infection (Khuroo et al. 2020). These vaccines consist of the inherent ability to induce toll-like receptors such as TLR 3, TLR 7/8 and TLR 9, components of the innate immune system that holds the involvement of B cells, CD4 and CD8 T cells. These are usually rapidly produced at a comparatively lower cost. However, broad adjunct testing is required for confirming their efficacy and safety. The post-vaccination mutation may be a result of viral replication, which might lead to the formation of recombinants (Kaur and Gupta 2020). (2) Inactivated vaccines, contained killed viruses; works in a similar fashion to the inactivated ones by triggering an immune response due to viral exposure (albeit dead). These are developed by inactivating viruses, mediated by formaldehyde or by heat. These are free of any living components of the virus. These vaccines do not require cold chains for distribution, it can be added simply by freeze-dry. These vaccines usually possess a suboptimal immune response, and they do not usually perform replication and are mostly used along adjuvants to enhance the immunogenic responses (Sanders et al. 2015). Since there lies a need to deal with a large number of viral species, it is very important to maintain their integrity; however, there are chances of Th2 cell skewed response. An example of an inactivated vaccine for mitigating COVID-19 is PiCoVacc, by Sinovac Biotech. (3) Protein vaccines: contains the protein directly administered via nanoparticles and triggers an immune response. The body then forms a memory of it for further infections and has antibodies prepared (Rawat et al. 2021). These consist of empty virus shells without any of their viral components and are believed to induce strong immune responses, apart from it being considered safe. However, they are considered difficult to manufacture (Loo et al. 2021). An example of these vaccines used to mitigate COVID-19 is Triple-Antigen Vaccine, by Premas Biotech. Apart, next-gen vaccines-nucleic acid vaccines have also emerged rapidly (van Riel and de Wit 2020), such as (a) DNA Vaccines: The vaccine of this category that is utilized to mitigate COVID-19 is INO4800, by INOVIO Pharma, Korean Institute of Health, and International Vaccine Institute. These vaccines consist of DNA that encodes the antigen from the specific pathogenic entity into a plasmid (antigenic components such as spike protein). These vaccines are considered safe and reportedly possess no risk of infection. These are considered to be highly immunogenic and when used along with inactivated vaccines, have been subjected to produce a high titer of neutralizing antibodies. The delivery process is aided with the help of an electroporation device. (b) RNA Vaccines: They are lipid-coated mRNA of the SARS-CoV-2 that aids the expression of spike proteins. Even though they have been proven to induce ADE, they are considered safe and have no cases of infections. Examples of these vaccines are mRNA-1273, by Moderna; and BNT162 (a1, b1, b2, c2), by BioNTech/Fosun Pharma/Pfizer. (c) Viral Vector Vaccines: Recombinant DNA Technology is incorporated for the design of these vaccines. The DNA which is inserted into the bacteria or the virus vector encodes for an antigen from the pathogenic entity (Keech et al. 2020). These vectors further express the antigens in these cells. These can be of replicating or of non-replicating types. Replicating vaccines consists of a few examples like Hepatitis B, HPV and pertussis. Non-replicating vaccines which mitigate COVID-19 are Ad5-nCoV by CanSino Biological Inc. The non-replicating vaccines are believed to induce long-term immunity. Comparative studies of the currently available vaccines are presented in Table 4.

**Table 4:** Comparative analysis of the currently available vaccines. *Sources*: The information summarized in the tables were extracted from the reports collected

Name	Type	Efficacy (%)	Dosage	Storage	Country/origin
Covaxin	Inactivated virus vaccine	81	2 doses with a gap of 28 days in between	+ 2–8 °C	Bharat Biotech (India)
BBIBP—CorV	Inactivated virus vaccine	72–86	2 doses with a gap of 21 days in between	+ 2–8 °C	Sinopharm (China)
CoronaVac	Inactivated virus vaccine	50.7–83.7	2 doses with a gap of 14 days in between	+ 2–8 °C	SinoVac (China)
JNJ-78436735	Viral vector vaccine	72	1 dose	+ 2–8 °C for short-term storage and − 20 °C for long term storage	Johnson and Johnson (USA)
Sputnik V	Viral vector vaccine	91	2 doses with a gap of 21 days in between	+ 2–8 °C for short-term storage and − 20 °C for long term storage	Gamaleya (Russia)
Novavax	Virus-like particle vaccine	96	2 doses with a gap of 21 days in between	+ 2–8 °C for short-term storage and − 20 °C for long term storage	(USA)
Covishield	Viral vector vaccine	70–82	2 doses with a gap of upto 12 weeks in between	+ 2–8 °C	Oxford/Astrazneca (UK and Sweden)
BioNTech/Pfizer	Encapsulated mRNA vaccine	96	2 doses with a gap of 21 days in between	+ 2–8 °C for 5 days and − 70 °C for longer duration	(USA and Germany)
Moderna	Encapsulated mRNA vaccine	94.1	2 doses with a gap of 28 days in between	+ 2–8 °C for short-term storage and − 20 °C for long term storage	USA

## Conclusions

Learning from the current pandemic, we can implement certain measures to prevent the adverse effects of pandemics in the future. By continuously identifying new sources of viruses and classifying them accordingly, we can predict future outbreaks and be prepared for them. Even though it is not 100% possible to predict all the ways a virus may evolve, it would still provide an edge over not having any preparation done. Hygiene and sanitation need to be at the forefront not only in personal but also in public domains. Routine health checkups and strict medical protocols need to be observed. It is important for the medical staff or laboratory to report any abnormal symptoms or new disease on time and not try to conceal the information due to fear of any backlash. Timely reporting would ensure quicker containment and a slower spread of the disease. The government needs to put the needs of the citizens before their own and put a halt to any social event that may involve a large gathering of people because even if all the participants are tested negative, the possibility of a false-negative test is always there and might lead to super-spreader event thus putting the entire population at risk. Any donations made at the time of a pandemic must be put to good use immediately to fund the resources that might be needed for an eventual second wave or any other mishaps. Only through severe preparedness, we can combat the challenges brought about by the pandemic.

## Data Availability

Contact authors.

## Abbreviations

COVID-19: Coronavirus disease 2019
DNA: Deoxyribonucleic acid
H1N1: Hemagglutinin 1 and neuraminidase 1/the influenza type-A virus
HCoV: Human corona virus
HPV: Human papilloma virus
MERS: Middle East respiratory syndrome
NGO: Non-government organization
ORF: Open reading frame
PPE: Personal protective equipment
RNA: Ribonucleic acid
SARS-CoV-2: Severe acute respiratory syndrome coronavirus 2
SAα2,3Gal: Alpha-2,3 sialic acid receptors
